# In Vitro Tracking of Human Umbilical Vein Endothelial Cells Using Ultra-Sensitive Quantum Dot-Embedded Silica Nanoparticles

**DOI:** 10.3390/ijms24065794

**Published:** 2023-03-17

**Authors:** Jaehi Kim, Sunray Lee, Yeon Kyung Lee, Bomi Seong, Hyung-Mo Kim, San Kyeong, Wooyeon Kim, Kyeongmin Ham, Xuan-Hung Pham, Eunil Hahm, Ji Yeon Mun, Mukhtar Anthony Safaa, Yoon-Sik Lee, Bong-Hyun Jun, Hyun-Sook Park

**Affiliations:** 1 Department of Bioscience and Biotechnology, Konkuk University, Seoul 05029, Republic of Korea; 2 Stem Cell Niche Division, CEFO Research Center, Seoul 03150, Republic of Korea; 3 School of Chemical and Biological Engineering, Seoul National University, Seoul 08826, Republic of Korea

**Keywords:** single-particle tracking, quantum dots, silica-coated QD-embedded silica nanoparticles (QD^2^)

## Abstract

The nanoscale spatiotemporal resolution of single-particle tracking (SPT) renders it a powerful method for exploring single-molecule dynamics in living cells or tissues, despite the disadvantages of using traditional organic fluorescence probes, such as the weak fluorescent signal against the strong cellular autofluorescence background coupled with a fast-photobleaching rate. Quantum dots (QDs), which enable tracking targets in multiple colors, have been proposed as an alternative to traditional organic fluorescence dyes; however, they are not ideally suitable for applying SPT due to their hydrophobicity, cytotoxicity, and blinking problems. This study reports an improved SPT method using silica-coated QD-embedded silica nanoparticles (QD^2^), which represent brighter fluorescence and are less toxic than single QDs. After treatment of QD^2^ in 10 μg/mL, the label was retained for 96 h with 83.76% of labeling efficiency, without impaired cell function such as angiogenesis. The improved stability of QD^2^ facilitates the visualization of in situ endothelial vessel formation without real-time staining. Cells retain QD^2^ fluorescence signal for 15 days at 4 °C without significant photobleaching, indicating that QD^2^ has overcome the limitations of SPT enabling long-term intracellular tracking. These results proved that QD^2^ could be used for SPT as a substitute for traditional organic fluorophores or single quantum dots, with its photostability, biocompatibility, and superior brightness.

## 1. Introduction

Recently, single-particle tracking (SPT) analysis was used to investigate the behavior of targets in live cells or tissues [1,2,3]. Fluorescent probes are required for this type of imaging using confocal laser scanning microscopy (CLSM) or total internal reflection fluorescence microscopy (TIRFM) [4,5,6]. Organic fluorescence dyes such as fluorescein isothiocyanate (FITC) or rhodamine b isothiocyanate (RBITC) have traditionally been used as SPT probes. However, these organic dyes have limited brightness, high photobleaching, and possess hydrophobic characteristics [7,8,9,10].

Quantum dots (QDs) are semiconductor nanocrystals that can emit a specific wavelength of light when stimulated by light. QDs can be fabricated to emit light of varying spectrums—from UV to infrared—by manipulating their sizes and shapes. The wavelength of light that stimulates QDs can also be varied in the same way [11,12,13,14,15,16,17]. QDs have a particle size ranging from 2 to 10 nm, which can readily be transported across the cell membrane to reach cellular organelles. The process can be accelerated by an hour of cell starvation and providing the cell with new growth or differentiation medium. QD has been widely utilized to label and trace vasculatures in various tissues and is a potential tool to investigate the pharmacokinetics of drug candidates because of their non-invasiveness, which allows continuous monitoring of vasculature [18]. In this regard, the response of endothelial cells must be elucidated upon direct exposure to QD. However, QDs are usually coated with hydrophobic ligands for colloidal stability during fabrication, allowing them to easily aggregate under physiological conditions. Furthermore, toxic metals such as cadmium, a significant component of the most widely used QDs, can cause cytotoxicity-related problems during bioimaging applications [19,20]. Even though encapsulation of single QDs can overcome these problems, it remains unsuitable for real-time SPT due to its blinking property [3,21,22,23].

Previously, we reported the fabrication of silica-coated QD-embedded silica nanoparticles (QD^2^) which a large number of QDs were embedded onto the surface of the silica template and encapsulated with silica shell [24]. Compared with single QD, QD^2^ has various advantages, including solid signal generation, low toxicity, and biocompatibility. In detail, QD^2^ emits a 200 times stronger fluorescence signal without significant blinking than the single QD owing to the number of QDs embedded. Moreover, the outer silica shell reduces cytotoxicity through prevents the leaching of cadmium ions into the external environment and enhancing biocompatibility with its hydrophilic property. Furthermore, because QDs of QD^2^ were protected from the outer environment with a silica shell, QDs of QD^2^ were not degraded or damaged by oxidation, and as a result, the photostability of QD^2^ was quite high [8,14,25]. Based on these properties, QD^2^ has been applied to various biological experiments, especially bioimaging, and improved bioimaging results were represented compared with single QDs [24,26,27].

In this study, fluorescence imaging of human umbilical vein endothelial cells (HUVECs) via the SPT method was tried by using QD^2^, which is a promising candidate for fluorescence probes of SPT with its excellent optical and biological properties. Optical properties and cytotoxicity against HUVECs of QD^2^ were analyzed before fluorescence imaging of HUVECs, and optimal concentration for imaging was found. After treatment of QD^2^ to HUVECs with optimized concentration, behaviors and cellular functions of QD^2^ uptaken HUVECs were observed and compared with those of native HUVECs via fluorescence imaging. To confirm the superiority of QD^2^ as a probe for SPT, a comparison with single QDs and long-term storage tests was also conducted.

## 2. Results and Discussion

### 2.1. Characterization of QD^2^

**Figure 1:**
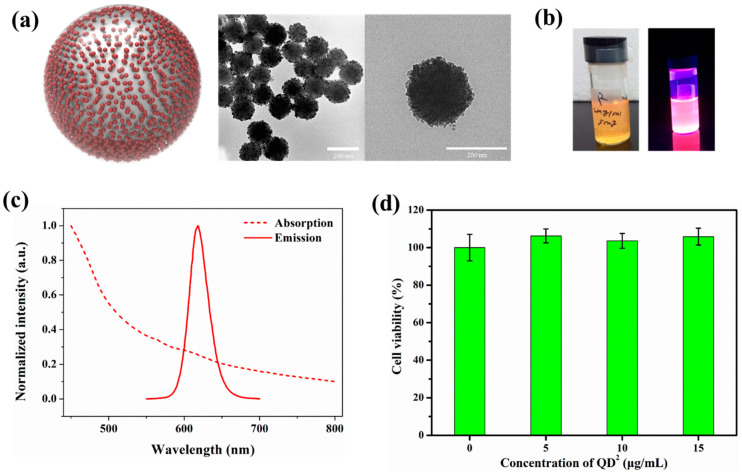
(**a**) Diagram of QD^2^ structure (left) and TEM images of QD^2^ (right). (**b**) Photograph of QD^2^ under visible light (**left**) and 365 nm UV light (**right**) (**c**) Excitation and emission spectrum of QD^2^. (**d**) Cell viability test of QD^2^-treated HUVECs.

QD^2^ was fabricated with purchased red QDs by using an established method (Figure 1a) [24]. As shown in Figure 1a, ca. 1000 red QDs were embedded onto the surface of silica nanoparticles (~180 nm) and encapsulated with a silica layer to increase biocompatibility and prevent leaking. Due to the excessive embedding of QDs onto the surface of the silica template, the fluorescence intensity of QD^2^ was sufficiently strong to be observed even with naked eyes under a UV illuminator (Figure 1b). The fabricated QD^2^ was excited when irradiated with shorter than 500 nm light and emitted 620 nm light with narrow full-width at half maximum (FWHM; Figure 1c). To evaluate the cell cytotoxicity of the fabricated QD^2^, HUVECs were treated with various concentrations of QD^2^ (5, 10, and 15 μg/mL) for 24 h and incubated with WST-1 for another 2 h. As shown in Figure 1d, QD^2^ showed negligible cytotoxicity at each concentration. Our results indicated that 15 μg/mL or less of QD^2^ was sufficient for cellular uptake in SPT of HUVECs without causing any cell cytotoxicity.

### 2.2. Cellular Uptake of QD^2^ for SPT

**Figure 2:**
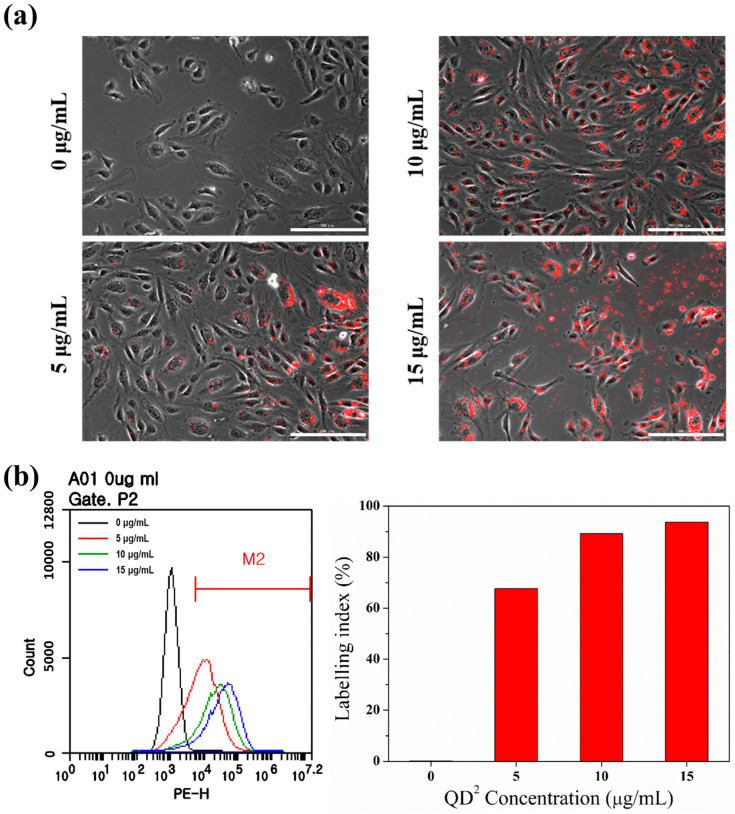
(**a**) Phase-contrast images (×20), scale bar size is 200 μm. (**b**) Flow cytometry analysis (**left**) and labeling index (**right**) of HUVECs with various concentrations of QD^2^ (5 μg/mL, 10 μg/mL, 15 μg/mL), 24 h after seeding.

Obtaining clear images in the SPT method warranted optimization of the conditions for cellular uptake of QD^2^. Various concentrations of QD^2^ (5, 10, and 15 μg/mL) were treated respectively to starved HUVECs for 24 h to make QD^2^ being uptaken into the HUVECs via clathrin-mediated pathway for endocytosis [28]. As shown in Figure 2a and Figure S1 in the Supplementary Materials, QD^2^-treated HUVECs showed a strong fluorescence signal, uniformly localized in the cytoplasm. Notably, the fluorescence signal of QD^2^ uptaken HUVECs was represented stably in real-time tracking (Movie S1 in Supplementary Materials). This stable fluorescence signal could be explained based on the structure of QD^2^. Because numerous numbers of QDs were embedded in the QD^2^, the fluorescence intensity of individual QD^2^ particles is stable unless embedded QDs blink simultaneously. The labeling index (LI) increased with increasing concentration of QD^2^; 67.7% at 5 μg/mL, 89.2% at 10 μg/mL, and 93.7% at 15 μg/mL (Figure 2b). Although the highest labeling efficiency was observed at 15 μg/mL, the labeling index reached a near plateau at 10 μg/mL, and the surplus unbound QD^2^ particles remained in the culture plate at 15 μg/mL. Therefore, we selected 10 μg/mL as an optimal concentration of QD^2^ for cellular uptake.

### 2.3. Retention of QD^2^

**Figure 3:**
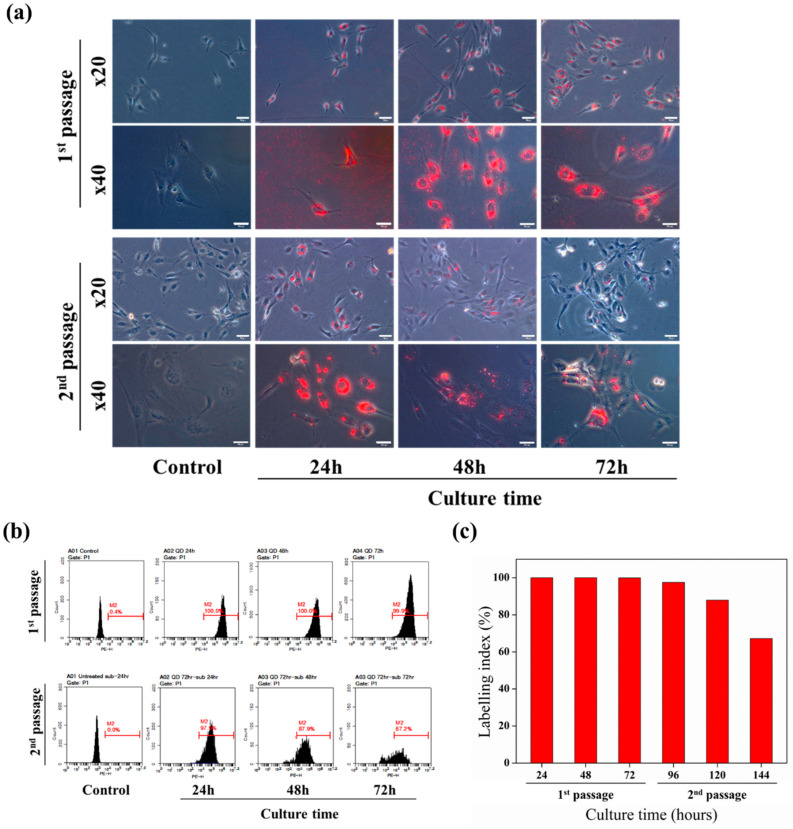
Extended retention of QD^2^ fluorescence at low cell density. (**a**) Phase-contrast images (Scale bar size; 20× = 75 μm and 40× = 5 μm) and (**b**) flow cytometry analysis of QD^2^-labeled cells for 72 h at 1st and 2nd passage. (**c**) Labeling index % of QD^2^-labeled cells for 72 h at 1st and 2nd passage. HUVECs were treated with 10 μg/mL of QD^2^ before passaging.

Retention of QD^2^ in HUVECs was investigated by using phase-contrast imaging and flow cytometry (Figure 3). Because QD^2^ might be secreted to the extracellular matrix (ECM) at high cell density, causing subsequent reduction of label retention, the initial cell density of HUVECs was adjusted to 5000 cells/cm^2^ [29]. As shown in Figure 3a, QD^2^ was located in the HUVECs of the first passage, whereas fluorescence appeared after 72 h in culture. The fluorescence signal from uptaken QD^2^ was also retained in the HUVECs of the second passage until 24 h, after which its intensity slightly decreased. Based on flow cytometry results, the LI of HUVECs was calculated to quantitively evaluate the retention of QD^2^ in HUVECs (Figure 3b,c). For the first passage HUVECs, LI was maintained at almost 100%. LI of HUVECs of the second passage was 97.5% 24 h after passage. However, it decreased to 87.9% and 67.2% at 48 and 72 h after passage, respectively. We hypothesized that the decrease in LI originated from the proliferation of HUVECs, leading to an increase in their numbers, whereas the amount of QD^2^ remained constant. As a result, LI related to the amount of contained QD^2^ in each HUVEC was decreased. These results indicated how the expiration time of QD^2^ labeling might be longer in differentiating or non-cycling cells than in proliferating cells.

### 2.4. Effect of QD^2^ Uptake on Cell Characteristics

**Figure 4:**
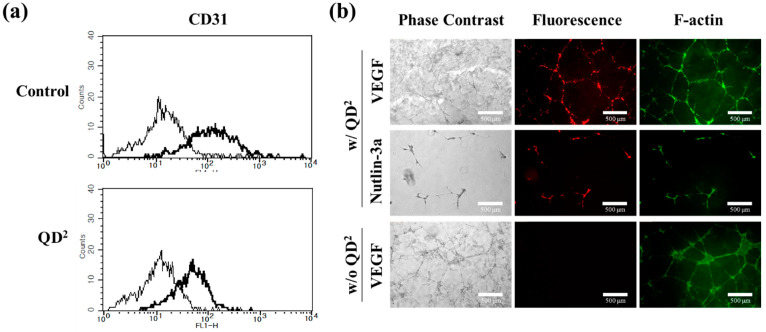
(**a**) Flow cytometry analysis of CD31 in native and QD^2^-labeled HUVECs. (**b**) Tube formation assay of control and QD^2^-labeled HUVECs. QD^2^ fluorescence enables following vessel formation in situ, overlapping with phalloidin staining. Treatment of angiogenic factor, VEGF (10 ng/mL), an angiogenesis inhibitor, and nutlin-3a (7.5 μM) reflects enhancement or breakdown of vessel formation (×20, scale bar = 500 μm).

To investigate the influences of QD^2^ treatments on cell characteristics, endothelial cell marker (CD31) expression and tube formation of HUVECs containing QD^2^ were investigated. As shown in Figure 4a, the average fluorescence intensity of QD^2^-treated HUVECs increased after the conjugation of the anti-CD31 antibody and FITC-conjugated secondary antibody, confirming the expression of CD31 on the cells. A similar pattern was also observed in native HUVECs, suggesting that the uptake of QD^2^ did not affect their CD31 expression. Figure 4b shows the fluorescence microscope images of in vitro tube formation assay with QD^2^-treated HUVECs and native HUVECs. Tube formation, representative of blood vessel formation and one of the unique characteristics of HUVECs, is enhanced when incubated with vascular endothelial growth factor (VEGF) and inhibited when incubated with nutlin-3a. Tube formation and inhibition of QD^2^-treated HUVECs could be observed via fluorescence imaging, and the tendency was the same with native HUVECs. Furthermore, the fluorescence signals from QD^2^ in HUVECs almost overlapped with those from F-actin, traditionally used for tube formation assays. These results supported that QD^2^ labeling did not impair the vessel formation of HUVECs, thereby aiding in the cell tracking of angiogenesis assays in real time.

### 2.5. Labeling Efficiency and Photostability of QD^2^

**Figure 5:**
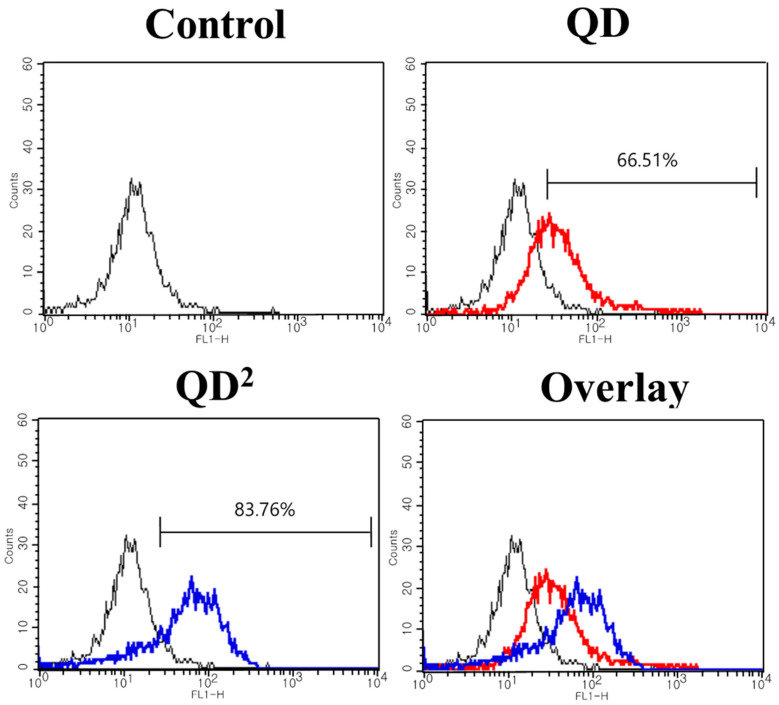
Flow cytometry analysis results of native (black line), QD-uptaken (red line), and QD^2^-uptaken HUVECs (blue line).

To compare the cell-loading efficiency of QD^2^ with a single QD, each HUVEC was treated with the same concentrations of QD and QD^2^ for uptake. Figure 5 represents the ratio of labeling of QD and QD^2^ after 24 h. The total QDs in QD^2^ were less than those in a single QD at the same concentration. However, QD^2^ showed a higher ratio of labeling than single QD (83.8% vs. 66.5%). The higher photoluminescence intensity of QD^2^ might be the cause of this higher labeling efficiency of QD^2^ compared with a single QD [24,27].

**Figure 6:**
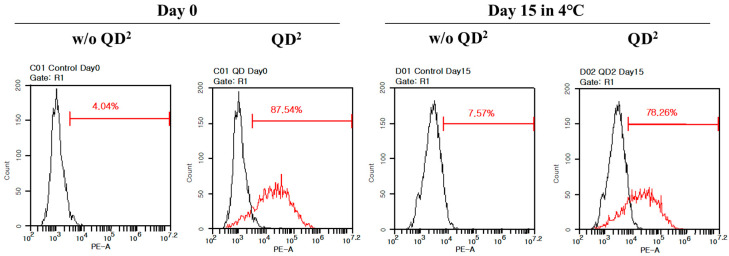
Flow cytometry analysis results of fresh QD^2^- and 15 days stored QD^2^-uptaken HUVECs. Black line represented native HUVECs, and red line represented QD^2^-uptaken HUVECs.

The photostability of QD^2^ was evaluated via a QD^2^ uptake experiment and flow cytometry. Fabricated QD^2^ was stored at 4 °C for 15 days and uptaken by HUVECs. As shown in Figure 6, the degree of detection was 87.5% when HUVECS uptook fresh QD^2^, whereas it was 78.3% for 15 days of stored QD^2^. Because the degree of detection only decreased by 9.2% even when QD^2^ was stored for 15 days, it was proved that QD^2^ had excellent photostability against long-term storage, favoring the advantage of using QD^2^ in the SPT method.

## 3. Methods and Materials

### 3.1. Materials

All reagents were used as received from the suppliers without further purification. Tetraethyl orthosilicate (TEOS), (3-mercaptopropyl)trimethoxysilane (MPTS), bovine serum albumin (BSA), phalloidin, β-glycerophosphate, L-ascorbic acid, and Alizarin Red S were purchased from Sigma Aldrich (St. Louis, MO, USA). Absolute ethanol (99.9%) and aqueous ammonium hydroxide (25.0–28.0%, NH_4_OH) were purchased from Daejung (Sihung-si, Gyeonggi-do, Republic of Korea). Dichloromethane was purchased from Samchun (Pyeongtaek-si, Gyeonggi-do, Republic of Korea). Phosphate-buffered saline (PBS, pH 7.4) and tris-buffered saline (pH 8.0) were purchased from DYNE BIO (Seongnam-si, Gyeonggi-do, Republic of Korea). Enzymatic solution (ADICOL) and CEFOgro™ENDO were obtained from CEFO Co., Ltd. (Seoul, Republic of Korea). Dexamethasone was purchased from Enzo Life Sciences (Farmingdale, NY, USA). Water-soluble tetrazolium salt (WST-1 reagent) was purchased from Daeil Lab Service (Seoul, Republic of Korea). Deionized water (DW) was produced using a Millipore water purification system of Vivagen (Seongnam-si, Gyeonggi-do, Republic of Korea). CD31 antibody was purchased from BD Pharmingen Inc. (San Diego, CA, USA). FITC-conjugated secondary antibody was purchased from Jackson ImmunoResearch (West Grove, PA, USA). CdSe@ZnS QDs in toluene (100 mg/mL) were purchased from Zeus (Osan-si, Gyeonggi-do, Korea).

### 3.2. Preparation of Thiol-Modified Silica NP Templates

Silica NPs with 150 nm diameter were prepared using the modified Stöber method [30]. Briefly, 1.6 mL of TEOS and 3.0 mL of NH_4_OH were mixed with 40 mL of absolute ethanol, and the mixture was vigorously stirred for 20 h at 25 °C. The fabricated silica NPs were centrifuged for 15 min at 8500 rpm and washed several times with ethanol. Then, 200 mg of silica NPs were dispersed in 4 mL of absolute ethanol, and 200 μL of MPTS and 40 μL of NH_4_OH were added to the mixture, stirring vigorously for 12 h at 25 °C. The resulting thiol-modified silica NPs were obtained by centrifugation and washed several times with ethanol to remove the remaining reagents.

### 3.3. Preparation of QD^2^

One mL of thiol-modified silica NP mixture (10 mg of NPs in 1 mL of ethanol) was mixed with 4 mL of dichloromethane. To this mixture, 50 μL of distilled water and 70 μL of QDs in a toluene solution (100 mg/mL) were added. The mixture was stirred vigorously for 20 min at 500 rpm. Then, 50 μL of MPTS and 50 μL of NH_4_OH were added to the solution, and the mixture was stirred for 6 h at 50 rpm. QD-densely embedded silica NPs were obtained after centrifugation and washing with ethanol at 7000 rpm. The washed NPs were dispersed in a mixture of 50 mL of absolute ethanol, 50 μL of TEOS, and 50 μL of NH_4_OH. This mixture was stirred for 20 h at 50 rpm, and the resulting mixture was centrifuged and washed with ethanol several times. The obtained QD^2^ was re-dispersed in absolute ethanol for storage. Before use, all ethanol was removed via centrifugation, and QD^2^ was re-suspended in water.

### 3.4. Characterization of QD^2^

Transmission electron microscopy (TEM) images of QD^2^ were taken using Carl Zeiss LIBRA 120 (Oberkochen, Germany). Ultraviolet-visible light absorption spectrum of QD^2^ was analyzed by using a UV-Vis spectrophotometer (Mecasys OPTIZEN POP, Daejeon, Republic of Korea). Photoluminescence (PL) intensity of QD^2^ was obtained by using a fluorescence spectrophotometer (Model Cary Eclipse, Agilent Technologies, Santa Clara, CA, USA).

### 3.5. Cell Culture and QD^2^ Labeling

By the Declaration of Helsinki, our research was approved by CEFO IRB Council (IRB approval number, CB-IRB-CD-120330). Human umbilical vein endothelial cells (HUVEC) were isolated from the umbilical cord of a healthy donor by ADICOL and cultured in CEFOgro™ENDO. Cells were starved for 1 h in basal media before QD^2^ labeling, which was then replaced with the growth medium with QD^2^ and incubated for 24 h at QD^2^ concentrations of 5, 10, and 15 μg/mL. After being washed, cells were cultured for live imaging, flow cytometry, and further passaging.

### 3.6. Cytotoxicity

To assess the cytotoxicity of QD^2^, a cell viability assay was performed using WST-1. HUVECs were seeded at a density of 5 × 10^3^ cells/cm^2^ and cultured for 24 h. Cells were treated with QD^2^ nanoparticles (5, 10, and 15 μg/mL) for 24 h. After that, 10 μL of WST-1 was added to each well and incubated for 2 h. Then, 100 μL medium was collected and transferred to a 96-well, and absorbance was measured at 450 nm by using a Cytation 5 Cell Imaging Multi-Mode Reader (BioTek, Winooski, VT, USA).

### 3.7. Cellular Imaging

To explore the extent of the QD^2^ label, a series of live-cell imaging was performed by taking a single snapshot of the cells every 15 min over 72 h. Each snapshot was taken at multiple points within a culture plate using Cytation 5 (Agilent Technologies Inc., Santa Clara, CA, USA). The cellular images were illuminated in citation 3 w/fluorescence microscopy module with Texas-Red filter (585/29 nm excitation, 628/32 nm emission).

### 3.8. Flow Cytometry

During passaging, 1 × 10^6^ cells were harvested separately from the successive passaging into 1.5 mL tubes and washed twice in PBS to remove the remaining growth media. These cells were then immediately run on a flow cytometer (BD Accuri C6 Flow Cytometer/BD FACSCalibur, BD Biosciences, Wokingham, UK). HUVECs, which had not been co-cultured with QD^2^, were used to gate cell population, and the percentage of HUVECs carrying QD^2^ intracellularly was analyzed.

To analyze the expressed surface proteins, cells were dissociated in cold PBS containing 1% (*w/v*) of BSA and washed twice. The cells were then incubated with a saturating concentration of CD31 antibody, placed on a shaker for 1 h, and washed three times with PBS containing 1% (*w/v*) of BSA. The samples were incubated with FITC-conjugated secondary antibodies for 30 min in the dark before being washed twice with cold PBS. Cells were analyzed by flow cytometry (BD FACS Calibur system) and the BD CELLQuest™ Pro program, version 5.1 (BD Sciences, Wokingham, UK).

### 3.9. Endothelial Cell Tube Formation Assay

HUVECs were seeded at a density of 25,000 cells/cm^2^ on Matrigel-coated plates, cultured for 24 h using CEFOgro™ENDO, and treated with 10 μg/mL of QD^2^ for 24 h. The QD^2^-labeled cells were supplemented with either 10 ng/mL of vascular endothelial growth factor (VEGF) (stimulator of angiogenesis) or Nutlin-3a (inhibitor of angiogenesis). After 24 h, the cells were stained with phalloidin for visualization and imaged using a fluorescence microscope (Leica, Wetzlar, Germany).

## 4. Conclusions

We successfully demonstrated a real-time cell tracking method with multiple QDs embedded in silica nanoparticles. The fluorescence intensity of QD^2^ was much higher than single QDs since ca. 1000 QDs were embedded in each QD^2^, preventing the blinking problems during bioimaging with single QDs. QD^2^ was not toxic against HUVECs and did not affect their innate characteristics, such as CD31 expression and angiogenesis capability. QD^2^ label was retained for 96 h in HUVECs, which is a proliferating cell type. The labeling efficiency of QD^2^ at 10 μg/mL concentration was significantly higher than that of QD at the same concentration (83.76% and 66.51%, respectively). Furthermore, the labeling by QD^2^ was retained even after storage for 15 days. These observations indicate the potential of QD^2^ to be used as an alternative probe for real-time cell tracking as they offer superior brightness, stability, and photostability compared to traditional organic dyes. Furthermore, their cost-effectiveness and potential for further performance improvements make them an attractive alternative to existing imaging technologies.

## Data Availability

All data generated or analyzed during this study are included in this manuscript and its Supplementary Materials.

## Supplementary Materials

The following supporting information can be downloaded at: https://www.mdpi.com/article/10.3390/ijms24065794/s1.

## Abbreviations

Single-particle tracking (SPT); quantum dots (QDs); silica-coated QD-embedded silica nanoparticles (QD^2^); confocal laser scanning microscopy (CLSM); total internal reflection fluorescence microscopy (TIRFM); fluorescein isothiocyanate (FITC); rhodamine b isothiocyanate (RBITC); reactive oxygen species (ROS); human umbilical vein endothelial cells (HUVECs); extracellular matrix (ECM); labeling index (LI); vascular endothelial growth factor (VEGF); tetraethyl orthosilicate (TEOS); (3-mercaptopropyl)trimethoxysilane (MPTS); bovine serum albumin (BSA); phosphate-buffered saline (PBS); water-soluble tetrazolium salt (WST-1); nanoparticle (NP).
