# Supplementary material for: In Vitro Tracking of Human Umbilical Vein Endothelial Cells Using Ultra-Sensitive Quantum Dot-Embedded Silica Nanoparticles
Source: Int J Mol Sci. 2023 Mar 17;24(6):5794. doi: 10.3390/ijms24065794 (PMC10052325; doi:10.3390/ijms24065794)
Supplement: Supplementary file 1 [file ijms-24-05794-s001.zip › Supplementary Materials Figure S1.pdf]

[Supplementary Material]

In Vitro Tracking of Human Umbilical Vein Endothelial Cells Using Ultra-Sensitive Quantum Dot-Embedded Silica Nanoparticles

Jaehi Kim [1,†], Sunray Lee [2,†], Yeon Kyung Lee [2], Bomi Seong [1], Hyung-Mo Kim [1], San Kyeong [3], Wooyeon Kim [1], Kyeongmin Ham [1], Xuan-Hung Pham [1], Eunil Hahm [1], Ji Yeon Mun [2], Mukhtar Anthony Safaa [2], Yoon-Sik Lee [3], Bong-Hyun Jun [1,*] and Hyun-Sook Park [2,*]

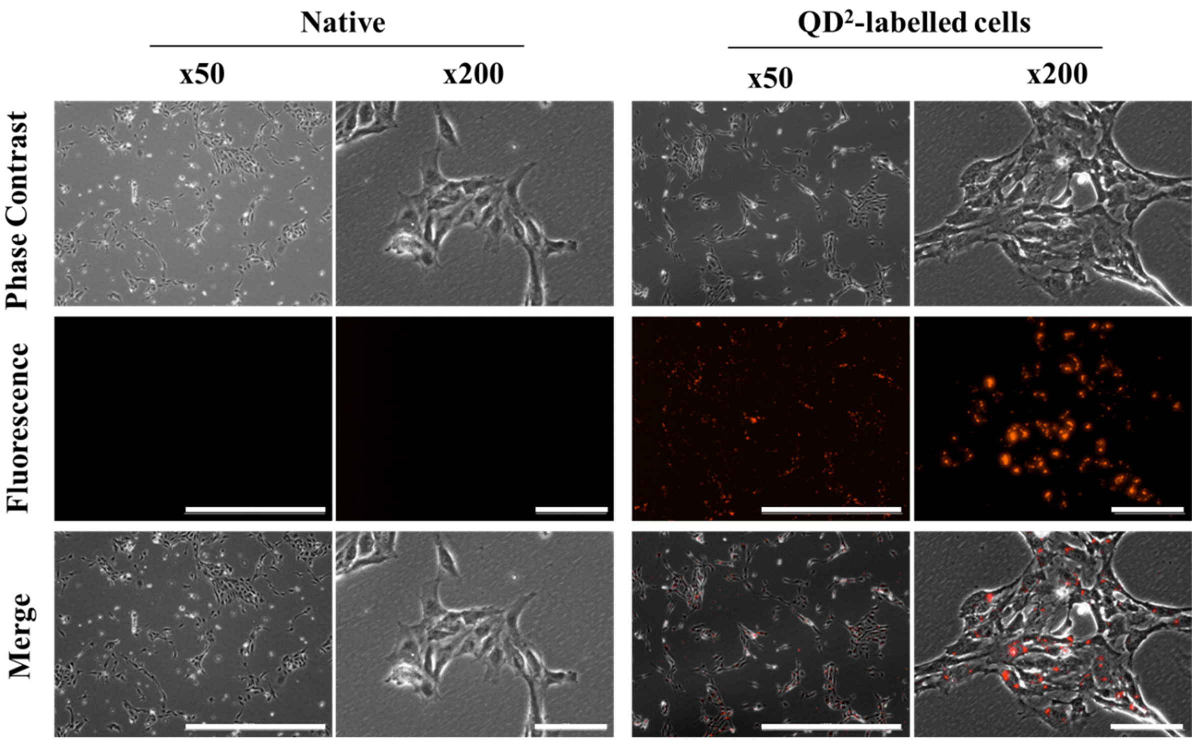

Figure S1. Phase-contrast images of HUVEC without and with QD^2 label (Scale bar size; 50X=1000 μm and 200X=100 μm).
