# Supplementary material for: In Vitro Tracking of Human Umbilical Vein Endothelial Cells Using Ultra-Sensitive Quantum Dot-Embedded Silica Nanoparticles
Source: Int J Mol Sci. 2023 Mar 17;24(6):5794. doi: 10.3390/ijms24065794 (PMC10052325; doi:10.3390/ijms24065794)
Supplement: Supplementary file 1 [file ijms-24-05794-s001.zip › Movie S1.pptx]

## Slide 1
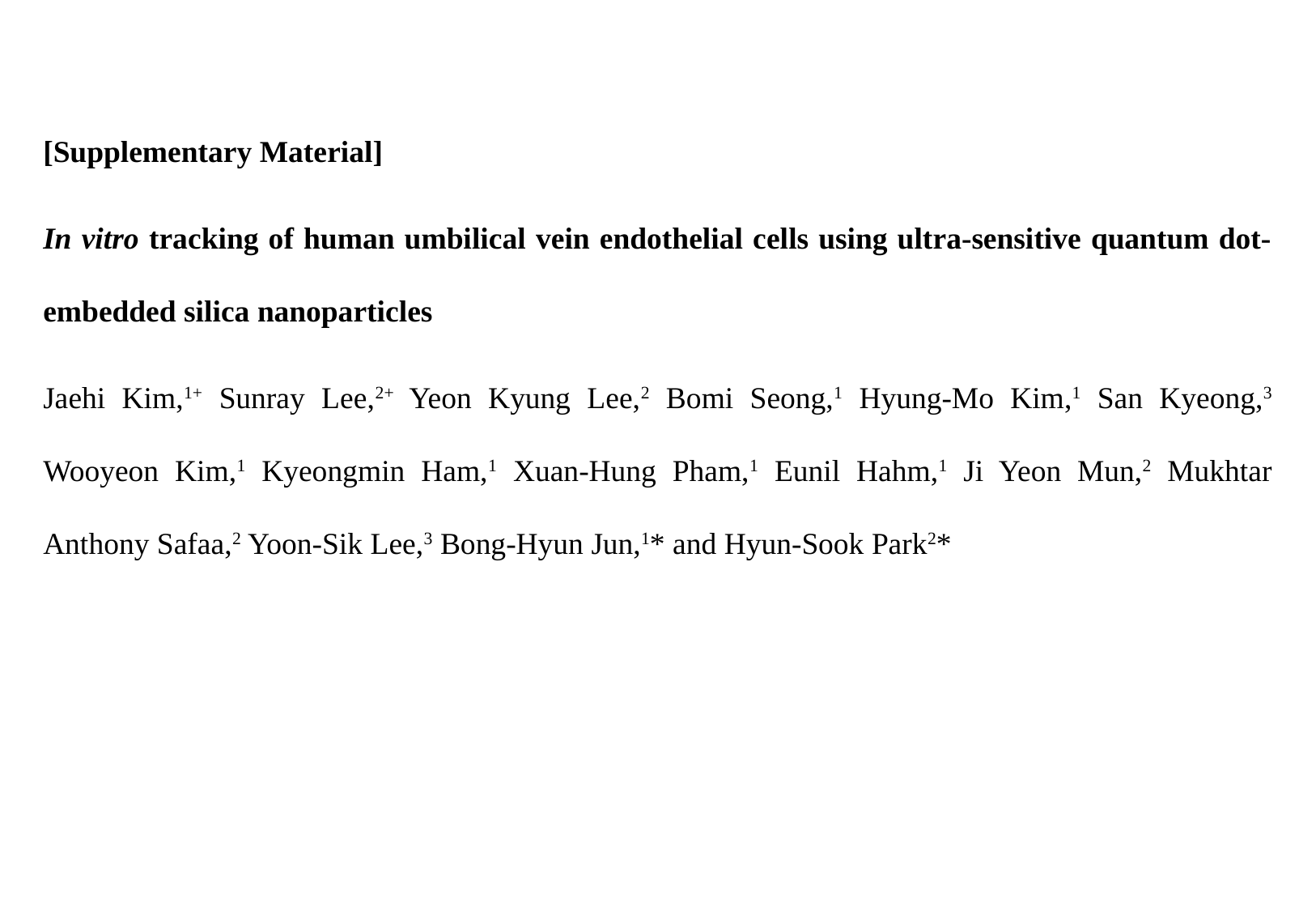

[Supplementary Material]
In vitro tracking of human umbilical vein endothelial cells using ultra-sensitive quantum dot-embedded silica nanoparticles
Jaehi Kim,1+ Sunray Lee,2+ Yeon Kyung Lee,2 Bomi Seong,1 Hyung-Mo Kim,1 San Kyeong,3 Wooyeon Kim,1 Kyeongmin Ham,1 Xuan-Hung Pham,1 Eunil Hahm,1 Ji Yeon Mun,2 Mukhtar Anthony Safaa,2 Yoon-Sik Lee,3 Bong-Hyun Jun,1* and Hyun-Sook Park2*

## Slide 2
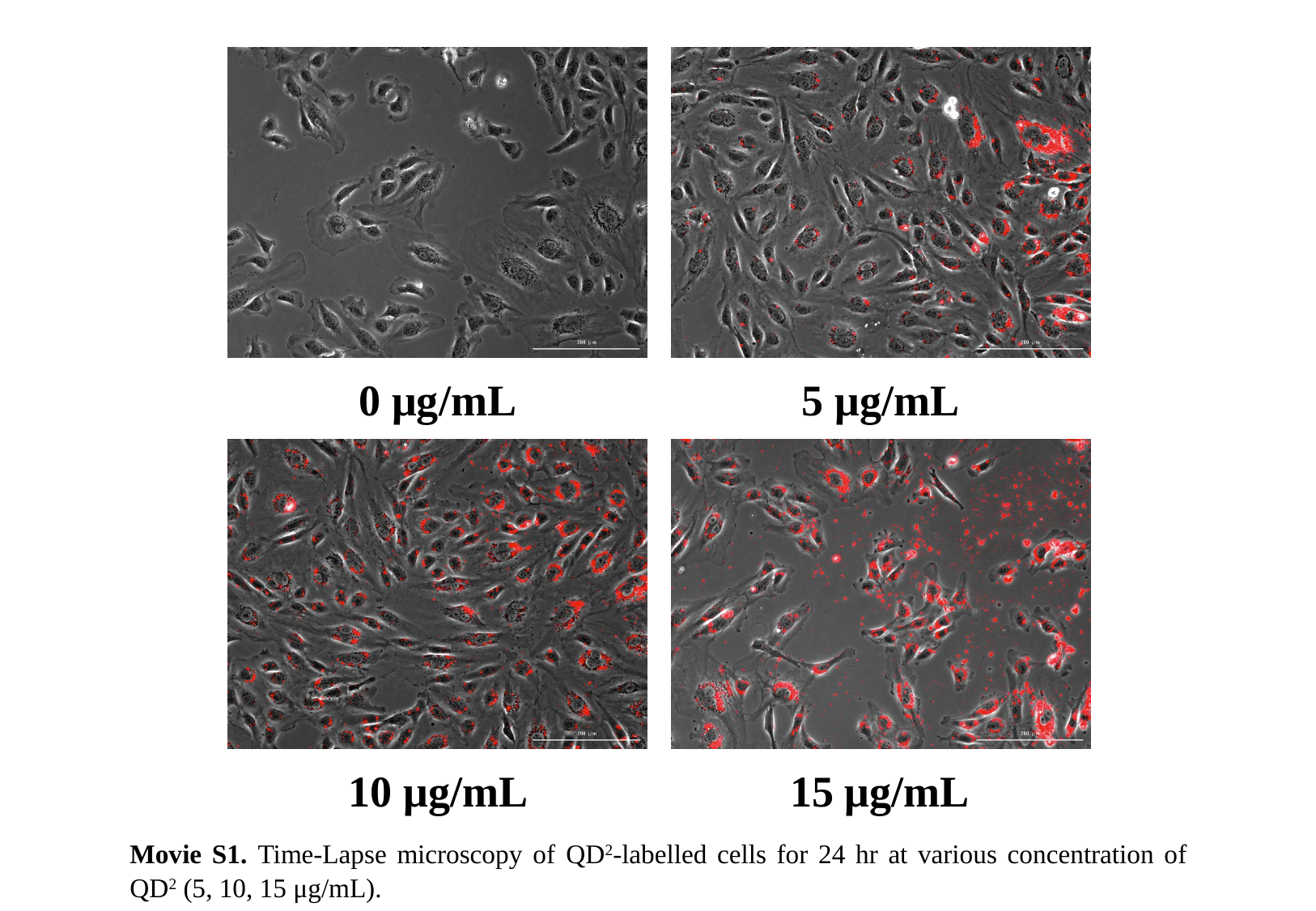

0 μg/mL
5 μg/mL
10 μg/mL
15 μg/mL
Movie S1. Time-Lapse microscopy of QD2-labelled cells for 24 hr at various concentration of QD2 (5, 10, 15 μg/mL).
